# Orbital Myositis in Pediatric and Adult Patients: A Comparative Case Report and Review of the Literature

**DOI:** 10.7759/cureus.114834

**Published:** 2026-08-20

**Authors:** Konstantinos Flindris, Athanasios Kaliardas, Konstantina Georgopoulou, Eleni Papafotiou, Marina Tsakiri, Vasiliki Gketsi, Ioannis Melissourgos, Ioannis Koumpoulis

**Affiliations:** 1 Ophthalmology, University General Hospital of Ioannina, Ioannina, GRC; 2 Ophthalmology, General Hospital of Ioannina G. Hatzikosta, Ioannina, GRC; 3 Pediatrics, General Hospital of Ioannina G. Hatzikosta, Ioannina, GRC

**Keywords:** case report, idiopathic orbital inflammatory disease, ioid, orbital myositis, orbital pseudotumor, pediatric orbital inflammation

## Abstract

Idiopathic orbital inflammatory disease (IOID) is a rare, noninfectious inflammatory disorder of the orbit and a diagnosis of exclusion. Orbital myositis, a subtype characterized by inflammation of one or more extraocular muscles, is uncommon in both children and adults and may mimic infectious, neoplastic, or systemic inflammatory conditions. We report two cases of orbital myositis in different age groups to highlight the variability in clinical presentation, diagnostic challenges, and management. An 11-year-old previously healthy girl presented with left periocular pain, eyelid edema, and conjunctival hyperemia. Initial treatment for presumed orbital cellulitis with oral and IV antibiotics failed, and the patient developed painful restriction of elevation and adduction. Computed tomography and MRI demonstrated enlargement of the left medial rectus muscle without sinus disease, abscess, or orbital mass. A 46-year-old previously healthy woman presented with headache, upper eyelid edema, binocular diplopia, and limitation of downgaze. MRI revealed enlargement of the left superior rectus muscle. In both cases, laboratory investigations excluded infectious, autoimmune, and systemic inflammatory etiologies. Prompt corticosteroid therapy resulted in rapid clinical improvement and complete resolution without recurrence. These cases illustrate the clinical spectrum of orbital myositis across different age groups. The pediatric patient initially mimicked orbital cellulitis, whereas the adult patient presented with painful ophthalmoplegia. Failure of presumed orbital cellulitis to improve with appropriate antibiotics, particularly in the absence of fever, sinusitis, or elevated inflammatory markers, should prompt consideration of orbital myositis. Characteristic imaging findings, including isolated extraocular muscle enlargement in the absence of sinus disease, abscess formation, or neoplasia, were crucial for diagnosis. Early recognition and prompt corticosteroid treatment generally result in excellent clinical outcomes, although biopsy and radiotherapy may be necessary in atypical or recurrent cases.

## Introduction

Idiopathic orbital inflammatory disease (IOID), historically referred to as orbital pseudotumor, is a benign, noninfectious inflammatory condition characterized by polymorphic inflammatory infiltration of orbital tissues in the absence of identifiable local or systemic causes [1]. IOID represents the third most common orbital disorder after thyroid eye disease and lymphoproliferative lesions, accounting for approximately 5-10% of orbital diseases encountered in clinical practice [2]. Although the condition predominantly affects adults during the fourth and fifth decades of life, pediatric cases have also been described and may exhibit distinct clinical characteristics [3].

The pathogenesis of IOID remains incompletely understood. Current evidence suggests an immune-mediated inflammatory process involving both cellular and humoral mechanisms. Histopathological findings vary according to disease stage and may include lymphocytic infiltration, plasma cells, eosinophils, fibroblast proliferation, and varying degrees of fibrosis [4]. The disease can affect virtually any orbital structure, including the lacrimal gland, orbital fat, sclera, optic nerve sheath, and extraocular muscles [5].

Moreover, orbital myositis is a specific subtype of IOID characterized by primary inflammation of one or more extraocular muscles and can follow either an acute monophasic or recurrent course. Although the rectus muscles are most frequently affected, any extraocular muscle may be involved, and the pattern of motility restriction depends on the location and extent of inflammation. It is considered the most frequent form of IOID in children and one of the most common presentations in adults [6]. Clinically, orbital myositis typically presents with acute orbital pain, painful eye movements, which are a characteristic feature, diplopia, periocular swelling, conjunctival injection, and restricted ocular motility. The disease is usually unilateral, although bilateral involvement may occur, particularly in pediatric patients and in association with systemic inflammatory disorders [7].

Radiologic imaging plays a crucial role in establishing the diagnosis. CT and MRI commonly demonstrate enlargement of affected extraocular muscles with involvement of their tendinous insertions, a feature that helps distinguish orbital myositis from thyroid eye disease, in which tendon sparing is more characteristic. Nevertheless, considerable overlap exists among various orbital inflammatory, infectious, and neoplastic conditions, necessitating a comprehensive diagnostic evaluation and proper disease management. No single laboratory biomarker is diagnostic; therefore, the diagnosis relies on the integration of clinical findings, orbital imaging, exclusion of identifiable infectious or systemic inflammatory causes, and the clinical course [8].

Herein, we present two cases of IOID presenting as orbital myositis in a child and an adult, highlighting diagnostic challenges, therapeutic approaches, and important considerations regarding biopsy and adjunctive treatment modalities.

## Case presentation

Case 1

An 11-year-old female was referred to our ophthalmology department due to progressive left periorbital inflammation associated with ocular pain and frontal headache. She had no relevant personal or family medical history and denied recent trauma, upper respiratory tract infection, sinus disease, or previous ophthalmic disorders.

The initial symptoms consisted of left periocular pain accompanied by mild swelling of the upper eyelid and conjunctival injection without fever. She was initially evaluated elsewhere and treated empirically with oral cefuroxime and topical tobramycin eye drops for presumed preseptal cellulitis. Despite 48 hours of treatment, her symptoms progressively worsened, prompting referral to our institution.

**Figure 1:**
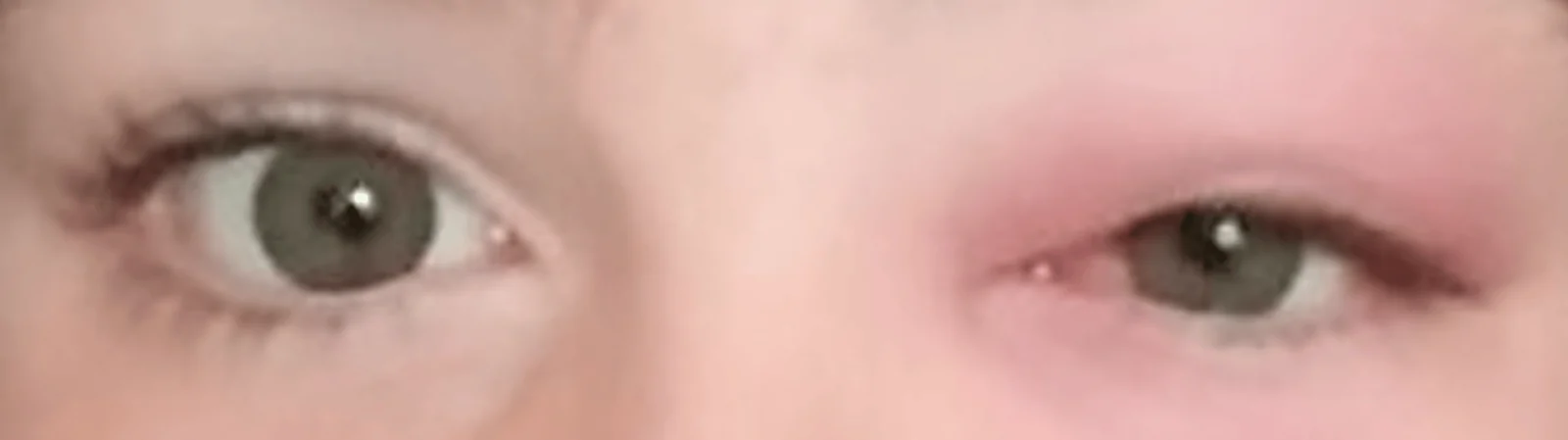
Clinical photograph at presentation showing marked left upper eyelid edema and erythema associated with mild mechanical ptosis, without apparent proptosis.

At presentation, best-corrected visual acuity (BCVA) was 20/20 in both eyes. Pupillary examination demonstrated equal and reactive pupils without a relative afferent pupillary defect (RAPD). Color vision and intraocular pressure (IOP) were normal bilaterally. External examination revealed edema of the left upper eyelid associated with mild ptosis (Figure 1). Slit-lamp examination demonstrated conjunctival hyperemia, circumcorneal congestion, and chemosis predominantly affecting the nasal bulbar conjunctiva. Hertel exophthalmometry did not demonstrate proptosis. Ocular motility was full in all directions of gaze, and no diplopia was reported. IOP measurements were within normal limits. Examination of the anterior segment and dilated fundus examination were unremarkable, with no evidence of anterior uveitis, optic disc edema, choroidal folds, retinal abnormalities, or posterior segment inflammation.

Extensive laboratory infectious, autoimmune, and endocrine investigations, including complete blood count with differential; erythrocyte sedimentation rate (ESR); CRP; renal function tests (serum urea and creatinine); liver function tests (aspartate aminotransferase (AST), alanine aminotransferase (ALT), alkaline phosphatase (ALP), and gamma-glutamyl transferase (γ-GT)); thyroid function tests (thyroid-stimulating hormone (TSH), free thyroxine (FT4), and free triiodothyronine (FT3)); thyroid autoantibodies (anti-thyroid peroxidase (anti-TPO) and anti-thyroglobulin (anti-TG)); antinuclear antibody (ANA); antineutrophil cytoplasmic antibody (ANCA); rheumatoid factor; serum angiotensin-converting enzyme (ACE); serum IgG4; and infectious screening for tuberculosis (interferon-gamma release assay) and syphilis (rapid plasma regain (RPR)), were unremarkable, and otorhinolaryngologic assessment excluded associated paranasal sinus disease. Orbital cellulitis was initially considered the most likely diagnosis; thus, the patient was admitted and treated with IV antibiotics (cefotaxime 2 g × 4 and clindamycin 40 mg/kg/day (560 mg × 4)).

**Figure 2:**
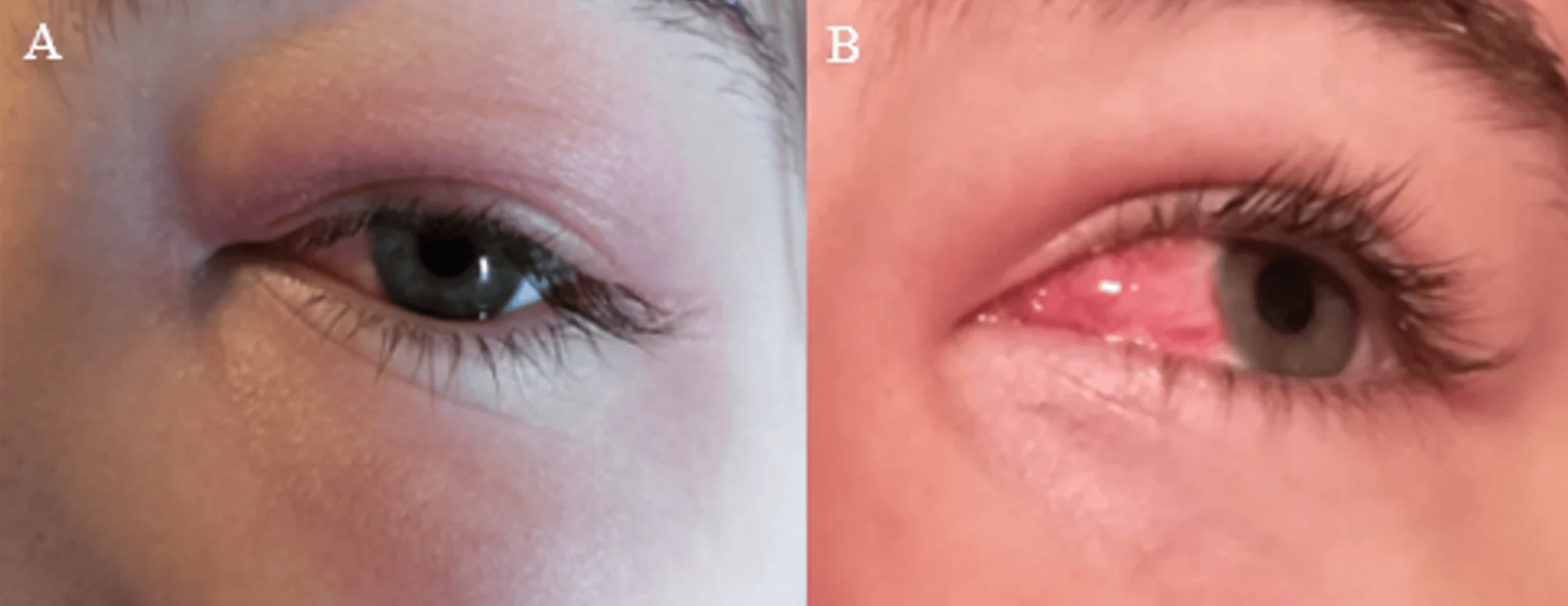
Clinical photographs (A, B) obtained after clinical deterioration despite 48 hours of IV antibiotic therapy. Marked progression of the left upper eyelid inflammatory edema and erythema is observed, accompanied by pronounced ptosis. The nasal bulbar conjunctiva demonstrates significant hyperemia and chemosis, particularly evident in left gaze. These findings were associated with the development of painful restriction of ocular motility, predominantly in elevation and abduction, raising suspicion for an inflammatory orbital process rather than infectious orbital cellulitis.

However, after 48 hours of broad-spectrum IV antibiotic therapy, the clinical condition deteriorated further. The left eyelid edema became more pronounced, conjunctival inflammation increased, and the patient developed painful restriction of elevation and abduction of the left eye, accompanied by new-onset diplopia in the corresponding fields of gaze (Figure 2). The absence of fever, normal inflammatory markers, lack of sinus involvement, and failure to respond to appropriate antimicrobial therapy prompted reconsideration of the diagnosis.

**Figure 3:**
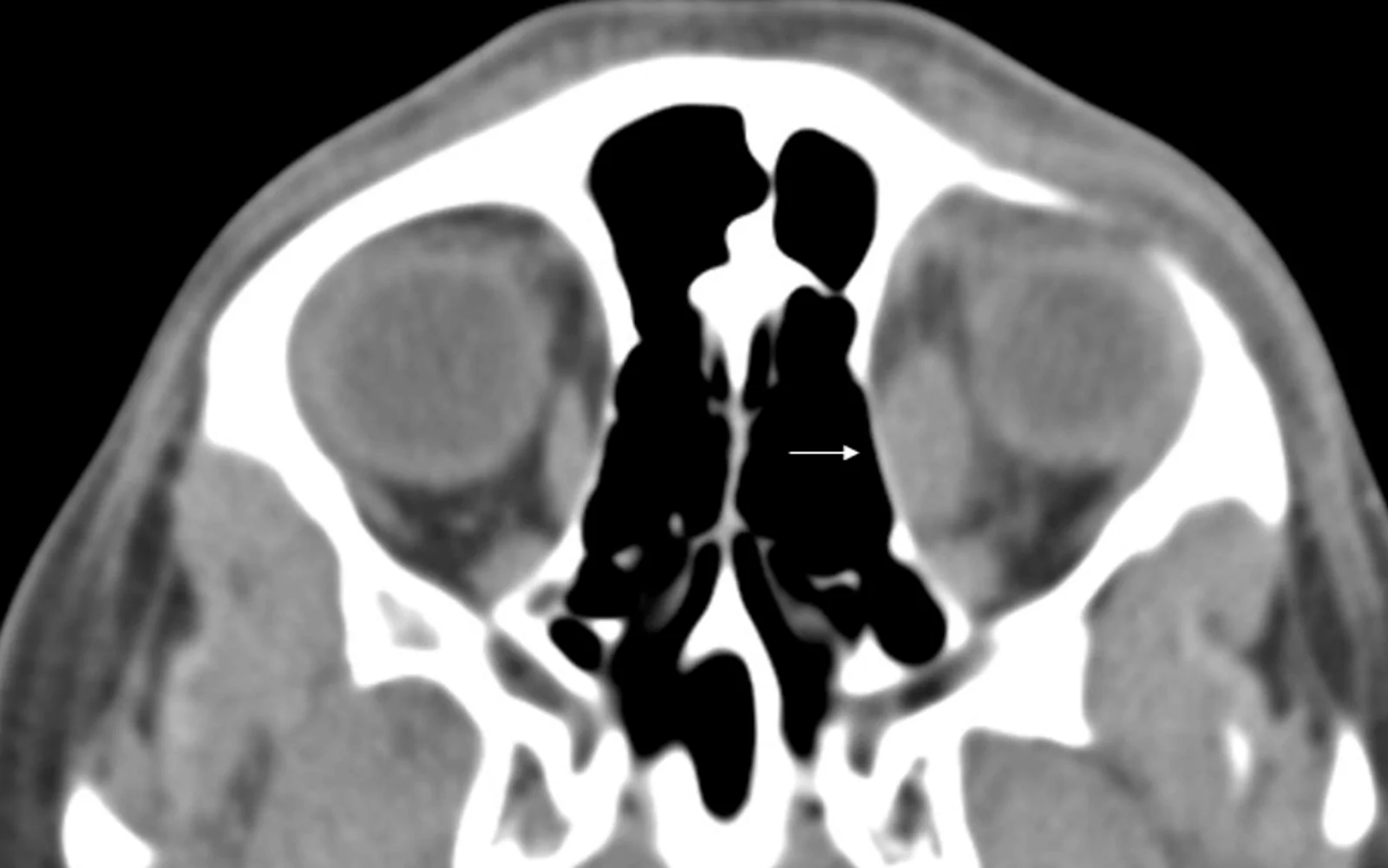
Coronal orbital CT demonstrating fusiform enlargement of the left medial rectus muscle (arrow) with associated mild inflammatory changes in the adjacent medial orbital soft tissues. No evidence of orbital abscess, orbital mass, bony destruction, or paranasal sinus disease is observed.

**Figure 4:**
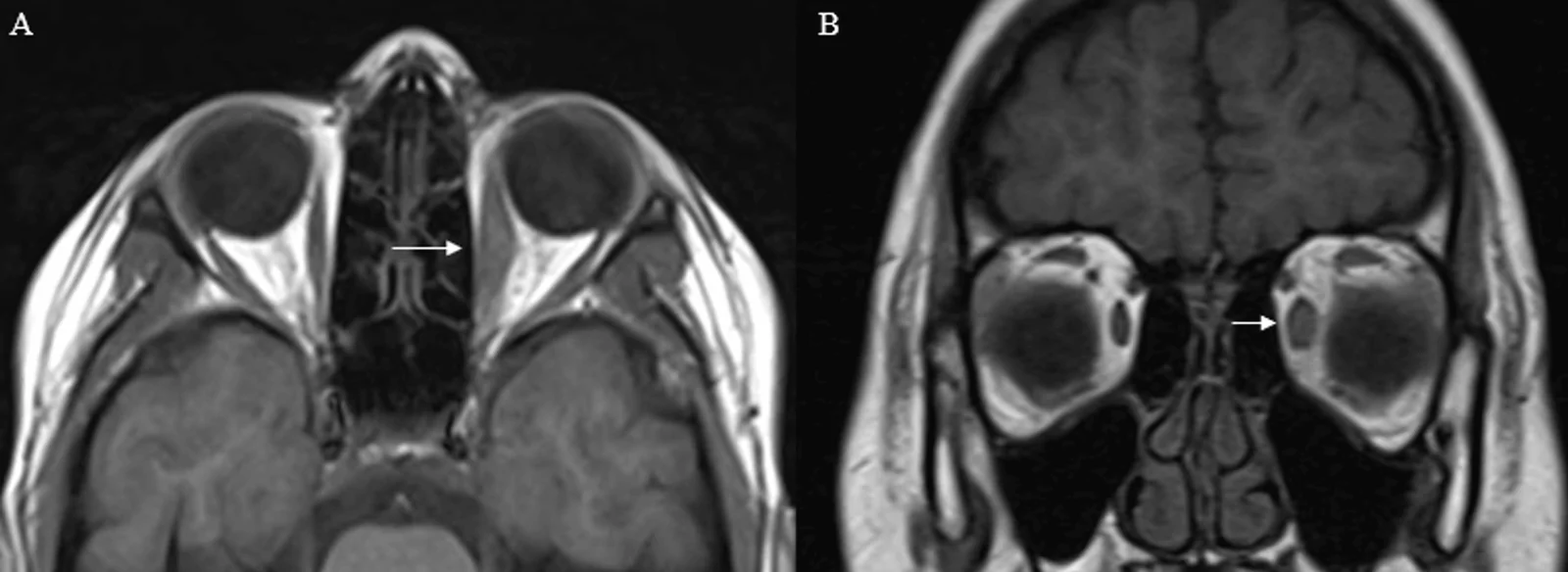
Orbital MRI (A) T1-weighted axial view and (B) T1-weighted coronal view, demonstrating fusiform enlargement of the left medial rectus muscle (arrows) with associated inflammatory changes of the adjacent orbital soft tissues. The thickness of the enlarged left medial rectus muscle was 7.4 mm, while the thickness of the right medial rectus muscle was 3.8 mm. No orbital abscess, optic nerve involvement, intracranial extension, or paranasal sinus disease is evident. The imaging findings were consistent with orbital myositis as a manifestation of IOID. IOID, idiopathic orbital inflammatory disease

CT of the orbits demonstrated left retrobulbar fat stranding and fusiform enlargement of the left medial rectus muscle associated with adjacent soft-tissue inflammatory changes, without evidence of abscess formation, bony erosion, orbital mass, or sinus disease (Figure 3). Orbital MRI subsequently confirmed enlargement of the left medial rectus muscle, including involvement of its tendinous insertion, together with inflammatory infiltration of the surrounding orbital soft tissues. The thickness of the enlarged left medial rectus muscle was 7.4 mm, while the thickness of the right medial rectus muscle was 3.8 mm. No optic nerve, cavernous sinus, or intracranial abnormalities were identified (Figure 4).

The absence of fever, normal inflammatory markers, lack of paranasal sinus disease, and failure to respond to previous antimicrobial therapy argued against an infectious etiology; however, orbital cellulitis could not yet be confidently excluded because the patient had marked progressive eyelid edema and erythema, prominent chemosis, painful restriction of ocular motility, and diplopia, while CT demonstrated retrobulbar fat stranding and adjacent orbital soft-tissue inflammatory changes. Because these clinical and radiologic findings may overlap with orbital infection, antimicrobial coverage was maintained during the diagnostic transition.

Clindamycin was discontinued, and antimicrobial therapy was escalated to IV vancomycin 60 mg/kg/day (900 mg × 4), while IV dexamethasone 0.2 mg/kg/day (3 mg × 4) was initiated for suspected orbital myositis. Vancomycin was selected to provide broader empiric Gram-positive coverage, including methicillin-resistant *Staphylococcus aureus*.

A significant clinical response was observed within 48 hours of corticosteroid administration. Ocular pain rapidly improved, eyelid edema and conjunctival inflammation decreased substantially, and ocular motility progressively recovered. Following stabilization, the patient was transitioned to oral methylprednisolone therapy with a gradual taper over six weeks. Complete clinical resolution was achieved without residual motility deficits, diplopia, visual impairment, or treatment-related adverse effects. No recurrence was observed at one-week, one-month, three-month, six-month, and 12-month follow-up.

The continued absence of systemic infectious features, normal inflammatory markers, absence of sinusitis or abscess on CT and MRI, characteristic isolated medial rectus enlargement with tendinous involvement, and failure to improve after broad-spectrum IV antibiotics collectively supported orbital myositis over orbital cellulitis.

Case 2

A 46-year-old woman presented to our ophthalmology department with a three-day history of progressive left-sided headache, upper eyelid swelling, and diplopia. Her past family and medical history were unremarkable. The patient described the initial onset of a dull frontal and periorbital headache localized to the left side, followed by progressive swelling of the upper eyelid. Within 24 hours, she developed binocular diplopia, particularly noticeable during downgaze. She denied fever, constitutional symptoms, or recent upper respiratory tract infection.

**Figure 5:**
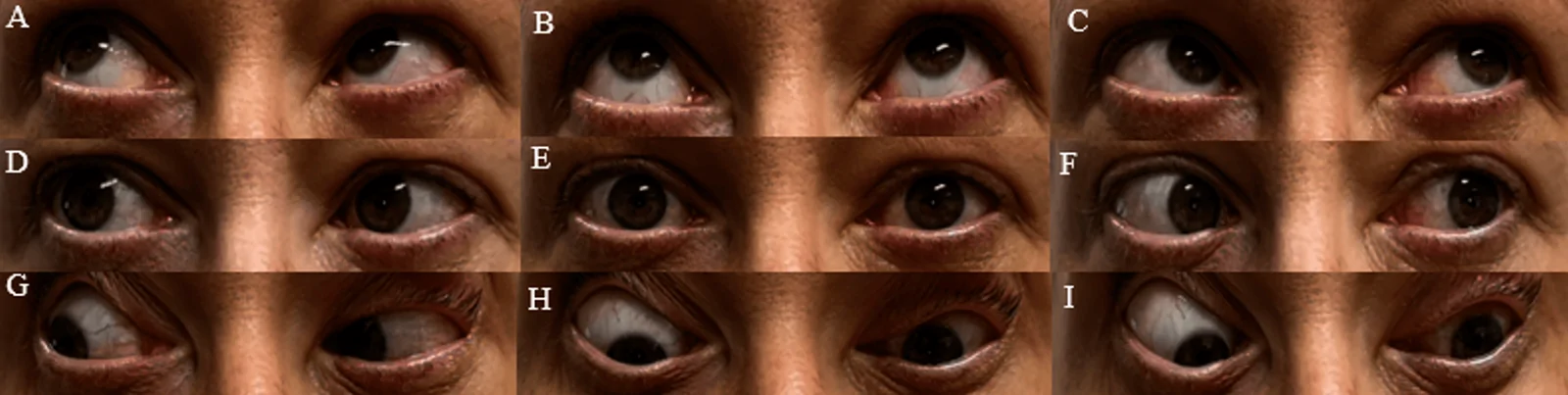
Nine-gaze ocular motility photographs (A-I), demonstrating limitation of depression of the left eye, producing an incomitant vertical deviation and binocular diplopia.

On presentation, BCVA was 20/20 in both eyes. Pupillary examination demonstrated equal and reactive pupils without evidence of RAPD. Color vision was preserved bilaterally. External examination revealed mild inflammatory edema of the left upper eyelid, and slit-lamp examination showed mild bulbar conjunctival injection without chemosis or anterior segment inflammation. Hertel exophthalmometry demonstrated no proptosis. Ocular motility examination revealed limitation of depression of the left eye associated with pain and diplopia during attempted gaze movements (Figure 5). The remainder of the ocular motility examination was normal. IOP was within normal limits. Dilated fundus examination was unremarkable, with normal optic disc appearance and no signs of retinal or choroidal pathology.

The differential diagnosis included orbital cellulitis, thyroid eye disease, IgG4-related orbital disease, granulomatosis with polyangiitis, sarcoidosis, lymphoma, and other infiltrative orbital disorders; hence, a comprehensive laboratory evaluation was undertaken to investigate potential infectious, autoimmune, endocrine, and systemic inflammatory causes of orbital inflammation, including complete blood count with differential; ESR; CRP; renal function tests (serum urea and creatinine); liver function tests (AST, ALT, ALP, and γ-GT); thyroid function tests (TSH, FT4, and FT3); thyroid autoantibodies (anti-TPO and anti-TG); ANA; ANCA; rheumatoid factor; serum ACE; serum IgG4; and infectious screening for tuberculosis (interferon-gamma release assay) and syphilis (RPR), which were unremarkable.

**Figure 6:**
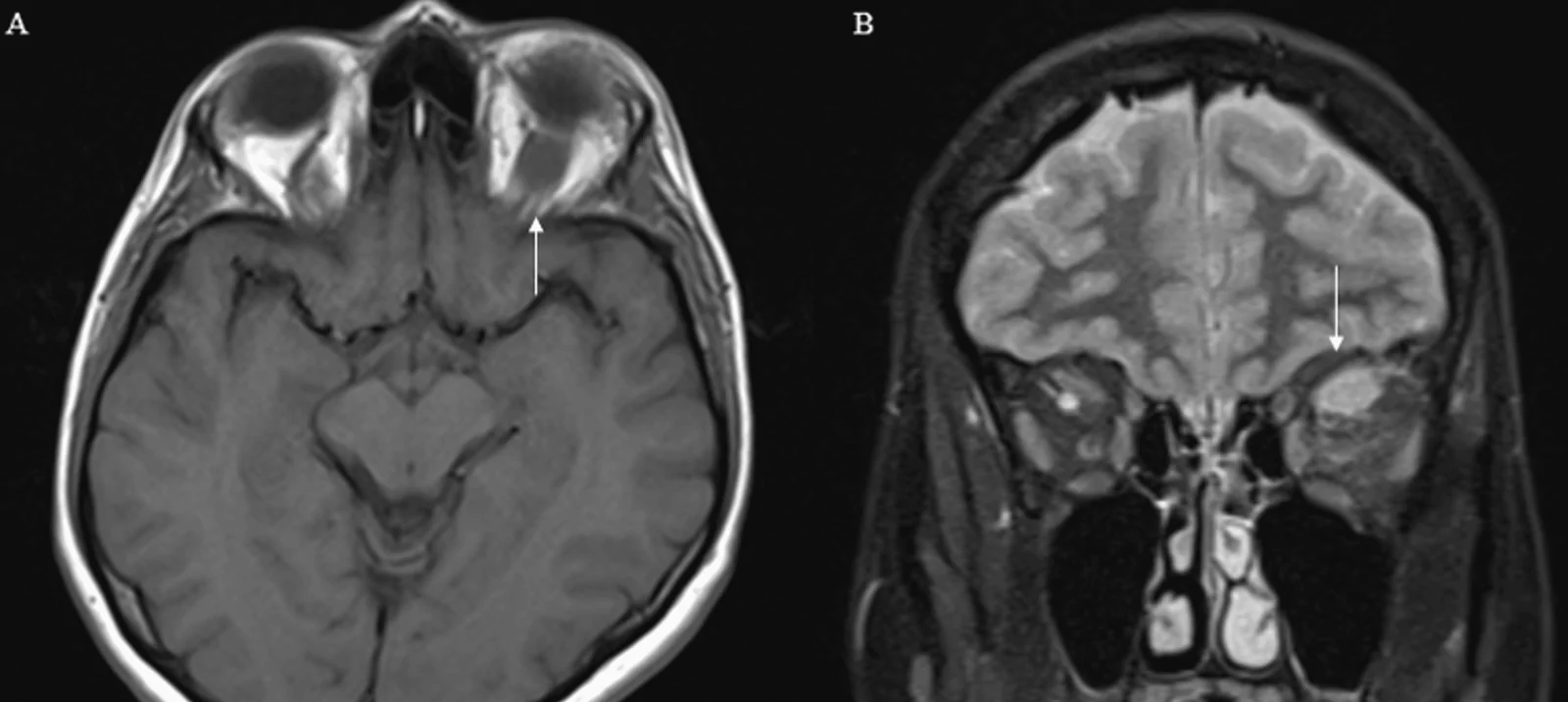
Orbital MRI (A) axial T1-weighted image and (B) coronal T2-weighted fat-suppressed image, showing fusiform enlargement of the left superior rectus muscle (arrows) consistent with active inflammation. The thickness of the superior rectus muscle was 7.8 mm, while the thickness of the contralateral muscle was 3.2 mm. No orbital abscess, optic nerve involvement, intracranial extension, or associated paranasal sinus disease was identified.

Orbital MRI revealed fusiform enlargement and contrast enhancement of the superior rectus muscle, including involvement of its tendinous insertion, with associated inflammatory changes in the surrounding orbital fat. The thickness of the superior rectus muscle was 7.8 mm, while the thickness of the contralateral muscle was 3.2 mm. The optic nerve, orbital apex, cavernous sinus, intracranial structures, and paranasal sinuses appeared normal (Figure 6).

The combination of acute painful ophthalmoplegia, characteristic radiologic findings, absence of sinus disease, negative systemic workup, and lack of evidence for neoplastic or endocrine pathology strongly suggested orbital myositis as a manifestation of IOID. Despite preserved visual acuity and color vision and the absence of RAPD, optic nerve involvement, or orbital apex disease, IV methylprednisolone pulse therapy was selected because of the rapid progression of painful ophthalmoplegia, functionally significant diplopia, and objective restriction of ocular motility over a short clinical course. The patient was admitted and treated with high-dose IV methylprednisolone 1,000 mg/day for three consecutive days. A marked clinical response was observed within 48 hours of treatment initiation. Headache and orbital discomfort rapidly improved, eyelid edema decreased substantially, and ocular motility progressively normalized. Diplopia resolved completely over the following days. Following the initial IV corticosteroid treatment, the patient was transitioned to oral methylprednisolone therapy with a gradual taper for four weeks. At subsequent follow-up examinations (one week, one month, three months, six months, and 12 months), visual acuity remained stable at 20/20, ocular alignment was orthophoric in primary position, and extraocular motility was full in all directions of gaze. No recurrence of symptoms or treatment-related adverse effects was observed during 12 months of follow-up.

The clinical course, diagnostic evaluation, and management of both cases are summarized in Table 1.

**Table 1:** Timeline of clinical course, diagnostic evaluation, and management.

Time point	Pediatric case (Case 1)	Adult case (Case 2)
Day 0	Ocular pain, frontal headache, upper eyelid edema, conjunctival hyperemia	Headache, upper eyelid edema, binocular diplopia
Days 1 and 2	Treated with oral cefuroxime and topical tobramycin for presumed preseptal cellulitis; no clinical improvement. Hospital admission; IV cefotaxime + clindamycin initiated	Best-corrected visual acuity 20/20; painful limitation of downgaze. Laboratory investigations negative
Day 4	Clinical deterioration despite antibiotics; painful ophthalmoplegia and diplopia. CT and MRI demonstrated isolated left medial rectus enlargement consistent with orbital myositis. IV dexamethasone initiated. Clindamycin changed to vancomycin	Orbital MRI demonstrated superior rectus enlargement consistent with orbital myositis. IV methylprednisolone 1,000 mg/day initiated
Day 6	Marked clinical improvement	Marked clinical improvement
Week 1	Transition to oral methylprednisolone taper	Transition to oral methylprednisolone taper
Week 6	Complete clinical resolution	Complete clinical resolution
Month 12	No recurrence	No recurrence

## Discussion

IOID, also known as idiopathic orbital inflammation or orbital pseudotumor, is a noninfectious, nonneoplastic inflammatory condition of the orbit diagnosed after exclusion of identifiable local or systemic causes [9]. Orbital myositis is a specific subtype in which one or more extraocular muscles are primarily involved [10]. The two present cases illustrate the age-related variability of this entity: the pediatric patient mimicked orbital cellulitis and deteriorated despite antibiotic therapy, whereas the adult patient presented with a more typical pattern of acute painful ophthalmoplegia and diplopia (Table 2). Both patients reported complete resolution of orbital pain and diplopia, returned to their usual daily activities without functional limitation, and were satisfied with their treatment.

**Table 2:** Comparative clinical and radiologic features of pediatric and adult orbital myositis cases.

Feature	Pediatric patient (Case 1)	Adult patient (Case 2)
Diagnostic challenge	Mimicked orbital cellulitis	More typical inflammatory presentation
Eyelid inflammation	Marked	Mild
Ptosis	Present	Absent
Chemosis	Prominent	Absent
Diplopia	Developed later	Present at onset
Muscle involvement	Medial rectus muscle	Superior rectus muscle
Radiologic findings	Medial rectus muscle fusiform enlargement	Superior rectus muscle fusiform enlargement
Tendinous insertion involvement	Yes	Yes
Clinical course	Progressive despite antibiotics	Early recognition
Corticosteroid response	Rapid and significant	Rapid and significant
Recurrence	No	No
Key message	Failure of antibiotic therapy should prompt consideration of orbital myositis	Orbital myositis should be included in the differential diagnosis of acute painful diplopia

The pediatric case is highly consistent with previously published pediatric IOID series. Spindle et al. reported 30 children with pediatric IOID, with a median age of 11 years, female predominance, unilateral disease in most cases, systemic symptoms in 40%, periorbital edema in 67%, blepharoptosis in 57%, and myositis on imaging in 33%. Recurrence occurred in 37% of cases [11]. Our 11-year-old patient closely resembles this cohort; however, she differed by having no systemic symptoms, no optic nerve involvement or uveitis, and no recurrence during follow-up, suggesting a localized and favorable type with an excellent prognosis rather than diffuse pediatric IOID (Table 3) [12-16].

**Table 3:** Comparative summary of published studies on pediatric and adult orbital myositis. EOM, extraocular muscle; IOID, idiopathic orbital inflammatory disease; NSAIDs, nonsteroidal anti-inflammatory drugs

Study	Population and design	Principal clinical findings	Treatment	Outcome and recurrence
Spindle et al. (2016) [11]	Multicenter retrospective series of 30 pediatric IOID patients	Periorbital edema 67%, blepharoptosis 57%, pain 40%, and reduced ocular motility 37%; initial misdiagnosis in 67%, commonly as cellulitis	Orbital myositis 33%; unilateral disease was predominant	Oral corticosteroids in 80%; selected recurrent cases received steroid-sparing therapy or additional treatment; complete resolution 83%; recurrence 37%, particularly among female and bilateral cases
Siatkowski et al. (1994) [12]	Retrospective series of 100 pediatric and adult patients	Muscle dysfunction could be normal, paretic, restrictive, or mixed, depending on disease stage	Orbital myositis with single-EOM involvement in 68%; lateral rectus muscle 33%; medial rectus muscle 29%	Early systemic corticosteroid therapy; steroid therapy response 68%; recurrence 15%; 9% subsequently developed thyroid eye disease
Mannor et al. (1997) [13]	Retrospective clinical and radiologic analysis of 26 pediatric and adult patients	Horizontal rectus involvement, multiple or bilateral muscle involvement, tendon sparing, absence of proptosis, and poor initial treatment response were associated with recurrence	Single acute episode of orbital myositis 77%; recurrent disease 23%	Systemic corticosteroids and/or nonsteroidal anti-inflammatory drugs; early systemic steroids proposed for patients with recurrence-risk features. Poor response to corticosteroids or NSAIDs was associated with recurrent or persistent disease
Yan et al. (2006) [14]	Retrospective series of 24 pediatric IOID patients	Palpable orbital mass, motility restriction, eyelid swelling, proptosis, and ptosis	Orbital myositis 8%; localized orbital mass and dacryoadenitis were more common	Systemic corticosteroids, surgery, radiotherapy, or combined treatment; complete recovery in 29%; overall treatment effectiveness 92%; one relapse (4%) successfully treated with radiotherapy
Belanger et al. (2010) [15]	Retrospective series of 12 children with idiopathic and secondary inflammatory orbital disease	Lacrimal gland enlargement, proptosis, painful ophthalmoplegia, and systemic symptoms in 50%	Orbital myositis 16.7%; bilateral disease was associated with systemic causes	Systemic corticosteroids; biopsy in refractory or atypical cases; significant response to corticosteroid treatment; recurrence documented in three patients (25%) with different inflammatory diagnoses
Kang et al. (2020) [16]	Retrospective series of 31 adult patients with idiopathic orbital myositis	Unilateral disease 84%; ocular motility limitation 71%	Medial rectus muscle most frequently involved. Multiple-muscle involvement was strongly associated with recurrence	Systemic corticosteroids; immunosuppressive therapy was used in selected recurrent cases; recurrence 35%. Rapid relapse after steroid discontinuation was associated with multiple recurrences
Ang et al. (2024) [17]	Multicenter retrospective MRI study of 24 adult patients with active idiopathic orbital myositis	Diplopia or restricted motility in 100%, orbital pain in 83.3%, and proptosis in 50%	Single-EOM involvement 75%; medial rectus most common. Active disease showed fusiform enlargement, high T2 signal, homogeneous enhancement, and frequent adjacent orbital fat inflammation	Systemic corticosteroids 91.6%; methotrexate and rituximab were required in one recurrent case; full resolution without visual sequelae in 62.5%; 37.5% had persistent or recurrent disease; prior recurrence predicted further episodes
Li et al. (2025) [18]	Retrospective imaging-based series of 10 pediatric IOID patients	Conjunctival injection 90%, optic disc edema 80%, visual impairment 80%, eyelid swelling 70%, and periorbital pain 60%	No isolated orbital myositis; predominantly diffuse disease, posterior scleritis, dacryoadenitis, or orbital-apex involvement	IV methylprednisolone followed by oral corticosteroid tapering; recurrence 90%, frequently during corticosteroid tapering; diffuse and deep disease appeared particularly relapse-prone

The diagnostic challenge in the pediatric case was the strong initial resemblance to orbital cellulitis. This overlap is well described in the literature, as IOID may present with eyelid swelling, erythema, conjunctival hyperemia, chemosis, pain, and motility restriction. Lee et al. similarly described a young patient initially treated for orbital cellulitis, in whom the absence of fever, leukocytosis, sinus disease, and lack of response to antibiotics prompted reconsideration of the diagnosis [19,20]. In our child patient, the lack of response after 48 hours of oral and IV antibiotics, normal inflammatory markers, absence of sinusitis, and subsequent imaging evidence of isolated medial rectus enlargement were decisive clues favoring orbital myositis.

The adult patient followed a more classical pattern: acute headache, eyelid edema, binocular diplopia, painful motility limitation, localized muscle enlargement, negative systemic workup, and rapid response to IV corticosteroids. Compared with the child, the adult case was less clinically suggestive of infection and was recognized earlier as an inflammatory myositis [16]. This comparison supports the main message of the case report: pediatric orbital myositis may present as presumed cellulitis with diagnostic delay, whereas adult orbital myositis more often presents as localized painful ophthalmoplegia.

Radiologic findings were similar in both cases and are consistent with the described imaging phenotype of idiopathic orbital myositis, which typically includes extraocular muscle enlargement, homogeneous enhancement, increased T2 signal on fat-suppressed sequences, and variable adjacent orbital fat inflammation [17,21].

Biopsy was not performed in either case because both patients had a typical presentation, characteristic imaging findings, no discrete orbital mass, no optic nerve or orbital apex involvement, negative infectious and autoimmune investigations, and rapid clinical response to corticosteroids [9,22]. This approach is supported by literature suggesting that biopsy is generally reserved for atypical presentation or imaging findings, discrete orbital mass, bone destruction, suspected IgG4-related disease, poor corticosteroid response within 24-72 hours, recurrence, progression, suspected malignancy, or nonmyositic disease. Recent consensus-based approaches also support histopathologic confirmation mainly in nonmyositic IOID, while prompt corticosteroid response is considered supportive in myositic IOID [23,24].

Nevertheless, the decision not to biopsy must be individualized. Boulter et al. emphasized that pediatric orbital inflammatory lesions have a broad differential diagnosis and may evolve diagnostically over time; they advocated biopsy before immunosuppressive treatment, particularly in orbital mass-like disease [25]. Therefore, in our pediatric case, biopsy was avoided because the lesion was isolated myositis, the infectious and systemic workup was negative, and the response to steroids was rapid and complete.

Corticosteroids remain the first-line treatment for IOID and orbital myositis. Both patients improved rapidly after IV corticosteroid therapy followed by oral tapering, which is consistent with reports showing rapid improvement within 24-48 hours in many pediatric and adult cases [26]. Pediatric reports recommend oral prednisone approximately 1-1.5 mg/kg/day with tapering over several weeks, while severe cases may receive IV corticosteroids initially. The rapid improvement in both patients supported the diagnosis and avoided unnecessary surgical intervention [27].

Radiotherapy was not indicated in either case because both patients had a first episode, preserved visual function, localized disease, and complete response to corticosteroids without recurrence. Radiotherapy is generally reserved for steroid-resistant disease, recurrent disease during tapering, steroid-dependent inflammation, contraindications to prolonged systemic corticosteroids, chronic fibrosing IOID, or incomplete response to immunosuppressive therapy [24]. Pediatric reports caution that low-dose radiotherapy should be considered only after exclusion of other causes and usually when corticosteroids fail, are contraindicated, or recurrence occurs despite treatment. In children, radiotherapy should be used especially cautiously because of long-term risks such as cataract, dry eye, radiation retinopathy, optic neuropathy, facial bone growth effects, and theoretical secondary malignancy risk [28].

The literature also highlights recurrence as an important issue. Spindle et al. reported recurrence in 37% of pediatric IOID cases [11], while more recent pediatric data suggest that diffuse and deep orbital subtypes may have even higher relapse rates and steroid dependence [18,29]. Our patients had no recurrence during available follow-up; nonetheless, long-term monitoring remains necessary, especially in the pediatric patient, because systemic inflammatory disease may emerge later and recurrence may occur after steroid withdrawal.

Overall, these cases contribute to the literature by providing a direct age-based comparison of IOID presenting as orbital myositis. The child demonstrates the diagnostic pitfall of presumed orbital cellulitis and the value of reconsidering the diagnosis when antibiotics fail. The adult demonstrates a more classic localized myositic presentation with diplopia and gaze limitation. Together, they emphasize that IOID should be suspected when orbital inflammation is painful, imaging shows extraocular muscle enlargement with tendon involvement, sinus disease and abscess are absent, laboratory workup is unrevealing, and the clinical response to corticosteroids is rapid.

The principal limitation of this report is the small sample size inherent to a case report. In addition, histopathologic confirmation was not obtained. Nonetheless, the characteristic imaging findings, exclusion of alternative etiologies, and rapid corticosteroid responsiveness strongly support the diagnosis of orbital myositis in both patients.

## Conclusions

Orbital myositis is an uncommon manifestation of IOID that may present with markedly different clinical features across age groups. The present cases highlight the diagnostic challenge of pediatric orbital myositis, which can closely mimic orbital cellulitis and lead to delays in diagnosis, as well as the more typical presentation of adult disease with painful ophthalmoplegia and diplopia. In both patients, characteristic radiologic findings were critical in establishing the diagnosis and excluding alternative infectious, neoplastic, and systemic inflammatory etiologies. The rapid and sustained response to corticosteroid therapy further supported the diagnosis and obviated the need for orbital biopsy. These cases emphasize the importance of maintaining a high index of suspicion for orbital myositis in patients with painful orbital inflammation, particularly when clinical deterioration occurs despite appropriate antibiotic therapy. Early recognition and prompt initiation of corticosteroids are essential to achieve favorable outcomes, prevent unnecessary invasive investigations, and preserve visual and ocular motility function.
